# Challenges and recommendations for addressing under-five pneumonia morbidity and mortality in Nigeria

**DOI:** 10.4314/ahs.v23i2.21

**Published:** 2023-06

**Authors:** Chidi okafor, Abimbola Olaniran, Elisabeth Darj

**Affiliations:** 1 Norwegian University of Science and Technology, Institute of Public Health and Nursing; 2 London School of Hygiene & Tropical Medicine, Department of Disease Control; 3 Norges Teknisk Naturvitenskapelige Universitet Institutt for Samfunnsmedisin, Institute of Public Health. and Nursing; Uppsala Universitet, women's and Children's Health

**Keywords:** Pneumonia, children, under five mortality, Nigeria, vaccinations, barriers

## Abstract

**Background:**

Pneumonia is a severe infection and one of the most common causes of mortality among children under five years of age, when not appropriately managed. Infection of the lungs by bacteria, viruses, or fungi and consequent inflammation may lead to cough and difficult breathing. Some of the key predisposing factors are malnutrition and air pollution. WHO reports that Africa has the highest burden of global child mortality, and 16% of all deaths in pneumonia, were children under five years of age in 2016.

**Objectives:**

This study aimed to explore how health providers perceive pneumonia as a cause of under-five mortality in Nigeria.

**Methods:**

A qualitative study design with in-depth interviews and focus group discussions was used to explore and understand nurses and pediatricians' views regarding the pneumonia situation, vaccinations, and preventive suggestions to reduce under five pneumonia deaths in Nigeria.

**Results:**

Two themes and four categories emerged: participant's anxiety over the situation, their views on impediments, current policies and strategies, and suggestions on addressing severe pneumonia.

**Conclusions:**

The results from this study highlight contextual issues playing major roles in pneumonia mortality among children in Nigeria, which will need approaches on several levels to address them.

## Introduction

Pneumonia is an infection, which inflames the lungs, caused by bacteria, viruses, or fungi, and may be enhanced by malnutrition, indoor and outdoor air pollution. This may be lethal if not appropriately managed, as the lungs are affected. The air sacs, alveoli, can be filled with fluid and obstruct oxygenation of the blood 1. In the early 20th century, there were few treatments for this disease and bloodletting was one of the recommendations 2. However, now w knowing the cause and, risk factors, there are available ways of prevention and treatment of the disease. Consequently, the number of children dying from pneumonia has decreased e due to interventions, such as vaccination, antibiotic treatment, reduction of major risk factors, reduction of childhood wasting, air pollution, and poor sanitation 3. Newly developed cheaper vaccines can be scaled up, which requires a surge of investment into research and roll-out programs. A study by the University of Southampton recently found that between 2000 and 2015, only 3 % of research funding dedicated to infectious diseases, was allocated to pneumonia prevention and treatment, which ignores its place as one of the leading causes of under-five mortality 4,5.

However, prevention and management of pneumonia among children U5 (under five years of age) is complex, the lack of investment and global initiatives, such as those seen for polio, malaria or HIV means that many pneumonia cases are misdiagnosed or mismanaged each year, and children do not survive 6.

Globally in 2004, 19% of child mortality were due to pneumonia, 17% to diarrhea 8% malaria, 4% measles, 3% injuries, 3% AIDS, and 8 % due to other neonatal infections 7.

Only five countries were responsible for more than half of child pneumonia deaths: Nigeria (162,000), India (127,000), Pakistan (58,000), the Democratic Republic of Congo (40,000) and Ethiopia 32,00 8,9. Despite the tools at our disposal, health care and antibiotic treatment, pneumonia still claimed the lives of more than 800,000 children U5 the last three year globally.

According to world poverty clock report in February 2019 over 91 million Nigerians live in extreme poverty and spend less than 1.90 USD per day. Compared to some other African countries, Nigeria has the largest proportion of people living in extreme poverty, while Tanzania, Kenya, South Africa and Zambia have about 19.9, 14.7, 13.8 and 9.5 million people respectively, living in extreme poverty 10. After the independence in 1960, Nigeria has become one of the countries with the highest incidence of poverty, making it difficult to reach the Sustainable Development Goal (SDG), number 1, to ‘End poverty in all its forms everywhere’. In spite of the measures initiated by the government this SDG has not been achieved. Thus, poverty is a major impediment to Nigeria's socio-economic development 11.

The picture of child mortality is similar in the rest of Africa. According to a WHO report, mainly Sub-Saharan Africa, has the highest burden of global child mortality. In 2016, 16% of the registered 5.9 million deaths in pneumonia were among children U5 12. The Nigerian government is implementing various health initiatives to improve public health, including the Integrated Management of Childhood Illness (IMCI) coordinated by WHO & UNICEF and aims to address the major clinical causes of U5 mortality. IMCI program has been training health workers to learn about the severity of the disease by observing the child for two key signs of pneumonia: chest in- drawing and fast breathing 13. The National Health Policy aims to reduce childhood mortality in Nigeria, and to enable all to achieve socially and economically productive lives. The National Primary Health Care Development Agency is responsible for developing and maintaining health care facilities across all local governments in the country, by trainings, updating the skills of the Primary Health Care (PHC) personnel, recruiting more village health workers, and traditional birth attendants with a goal of reducing child mortality in the country 14. Additionally, awareness campaigns have been launched on common childhood diseases and vaccinations, which are provided at no cost for the children in the country. Thus, the government is addressing the issue of child pneumonia and puts efforts and resources to minimize fatal outcome of this disease.

Nigerian U5 children had the highest number of pneumonia deaths in 2018, which should not be accepted Major risk factors for child pneumonia are known, inequalities in society remain. Children born in the poorest homes are four times more likely to die before the age of five, compared those born into wealthy families 15. These deaths should be prevented through simple and cost-effective interventions.

The data in Table 1 show that more effort is needed to reduce the mortality rate and urgent to provide concrete strategies to reduce child pneumonia death in several African and Asian countries 7.

**Table 1:** Estimated year Nigeria and five other countries are expected to reach the 2025 Global Action Plan for Pneumonia and Diarrhea, target at current rate of progress by WHO 2018 (7)

Country	Number of under-five death from pneumonia	Rate of under-five pneumonia deaths per 1000 births	Share of death from pneumonia in under five mortalities	Average annual rate of reduction in pneumonia mortality from year 2000 to 2015	Year country is expected to reach the 2025 GAPPD target at current rate of progress
Nigeria	132,556	19.0	17.8%	-2.73%	2075
India	178,717	7.1	14.9%	-5.76%	2030
Pakistan	63,844	11.9	14.8%	-3.72%	2052
Republic of Congo	45,812	14.8	15.2%	-1.75%	2075
Ethiopia	31,456	10.0	17.4%	-2.79%	2032
Niger	18,247	19.2	20.8%	5.53%	2048

## Methods

### Study design

A qualitative study design with in-depth interviews and focus group discussions (FGDs) was used to explore nurses and pediatrician perception of the pneumonia situation among children, access and use of vaccinations, impediments, and resolution in fighting pneumonia among children U5 in Nigeria.

### Study setting

The study was conducted in six different geo-political zones; North Central, North East, North West, South East, South South, and South East. The largest ethnic groups are Hausa, Igbo, and Yoruba. Government teaching hospitals, primary health centers and private hospitals in urban and rural areas in the selected states were included to get a purposeful sample of participants from various regions. This study was conducted between April 2021 and June 2021.

### Study participants

In total 32 nurses (N) and 6 pediatricians (PED) were interviewed. Eighteen nurses were included in three FGDs and 14 nurses were individually interviewed. The interviewees were both men and women within the age range of 38 and 54 years of age. Three nurses were interviewed in each state, one nurse from a private hospital (PH), one in a primary health center (PHC), and one from a Government Teaching Hospital (GTH), respectively. One pediatrician was interviewed at each teaching hospital. Participation was voluntary, they could leave at any time without giving a reason. Only nurses and pediatricians with a minimum of six years working experience with children were recruited for the research.

### Data collection

The first author visited the health facilities and recruited voluntary participants that showed interest in the research and met the inclusion criteria. Date and time were set for the interviews and FGDs respectively. The participants were informed of the research objectives and aims prior to the commencement of the interviews and discussions. The FGDs varied between 45 and 60 minutes and were conducted in conference centers in a hotel. The individual interviews were performed through telephone or physically. The interviews were conducted in English, local dialect, and Pidgin English. This was to enable the participant to communicate and understand the interviewees properly, as the interviewer speaks all the languages. Permission for recording the dialogues were obtained from the participants before start. The participants' quotes are coded with the number of the cited participant and by profession, nurse or pediatrician, and by the health facility they were working.

### Data analysis

We performed manifest content analysis following Graneheim and Lundman to understand the meaning of the transcriptions 16. The data was transcribed from the interviews and FGDs in local and pidgin English and translated into English. The transcriptions were consistently read to enable the researchers to become familiar with the context and to identify their inherent value. Meaning units were identified, condensed and coded. The process was continued until agreement on subcategories, categories and themes were obtained by the authors. Mindjet's mind manager version 2019 was beneficial to visualize the data coding and during the analysis 17.

### Ethical approval

The protocol was approved by the host institution NTNU, the Regional Ethic committee in Mid Norway (nr 147793) stated as no personal health information from the participant would be asked for, and no approval was needed. The local ethical committee in Nigeria also approved the research before the commencement of the study. Audio and visual privacy of the participants were maintained, by conducting the interviews in an environment, with no other people listening. The data material was only available for the researchers.

## Results

### Challenges

#### Anxiety of the current situation

The health workers shared their concerns regarding the preventable loss of children due to pneumonia. As vaccines are available to prevent pneumonias well as resources for diagnosis and treatment, nurses and doctors were much concerned that children were dying unnecessarily. They expressed helplessness and sometimes felt hopeless when they were unable to save the child's life. They talked about poverty, lack of knowledge, culture and religion affecting the situation. The parents, often the mothers, came too late to the health facility. Lateness was explained by parents searching for other, less expensive, treatments before turning to the health sector, and women waiting for the husband's approval before taking the child to a clinic for treatment. The health workers highlighted that decision making rest solely on the husbands, but 206 care seeking was most often accomplished by the wives. Cultural norms compel women to identify problems/illness and notify their husbands for approval, before acting regardless of the urgency of the situation.

#### Children are dying unnecessarily

I lost count of pneumonia patient we always admit here. The mothers use different available medium to treat their children, when their effort fails, then you see them here. No mother wants to watch her child die. 3N, GTH (Government Teaching Hospital)

When we ask them why they waited that long till the situation of the child got worse before coming to the center…most of them reply, it's my husband that dictates when to visit or not visit the center. 1N, PHC (Primary Health Center)

#### We are helpless

I once attended to a mother that lost her baby to pneumonia. She said the only reason she considered a traditional healer was because her husband said there was no money to take the child to the hospital. But when she was not seeing any improvement, she started begging for money from friends, but when she eventually came to the hospital, it was too late because the situation was complicated already, so the baby died the same day. 3N, GTH

I am just three weeks old in this center. I don't think I will continue after one month here. I can't stand the way children are dying out of ignorance of their parents. People in this community believe so much in herbs… I can't continue witnessing daily death. As a mother, I find it difficult sleeping at night after work. Preventable diseases like pneumonia should never take life again. 11N, GTH

#### Impediments

Health care providers from different states and health sectors explained how poverty, illiteracy, culture, and religion indirectly affected the outcome of pneumonia. They narrated how children were dying daily because of the negligence of the parents. Pneumonia was highlighted as an unnecessary cause of death and impediments were addressed. Even though there was no specific topic in the interview guide focusing on poverty or illiteracy, these perceptions came up in every discussion. The culture to use local herbs, visiting healers and unqualified kiosk keepers were known to the health providers. Religion was also seen as having an impact on decision making and women's access to health care. They mentioned the family's faith in Allah was another cause of why they turned up too late to a health facility. Allah decides on death, sickness as punishments for sin committed by their parents. Others believe in fasting and prayers to cure diseases without taking vaccinations or medications. The health providers discussed the reasons that most husbands stop their wife from making use of the available facilities, because of their financial inability to foot medical bills, coupled with their low level of education. In rural communities it was said that some farmers were killed, which increased poverty and hunger and made it difficult for the rest of the family to afford medical care for their children.

#### Poverty and illiteracy

When health issues become complicated, the bill goes up, this is just the fact. 2PED, GTH.

I put the blame on the husbands…I don't think they just stop their wife from accessing the hospital just because they derive pleasure in doing that. It's either because they don't know the importance or because they don't have the financial ability to pay for hospital bills. It can even be difficult to afford transportation. 2N, PH (Private Hospital) Most of the mothers that visits our health center don't know either the cause of pneumonia or how to prevent it. I will even say 99 percent of them don't have a clue. 30N, PH

#### Culture and religion

There is a medicine called Okwume in our dialect, which is what majority of the mother use in this community for any related pneumonia symptoms. They claim it takes care of everything vaccination will do. Only few people bring their babies for vaccination. 17N, PHC.

It's so strange to comprehend that someone will want to treat a dying child with fasting and praying. I have seen a lot in this community. I am a spiritual man, but my spiritualism does not stop me from treating my kid. I wish I understand where they got that concept of praying from. 2PED, GTH.

That sickness came from Allah but only few remember to keep calling on Allah when the situation of the child gets worst. It's when the child is about to die that they give you audience and expect you to perform magic on their child…most of the time, it's too late to do anything. 3PED, GTH.

#### Malnourishment and indoor cooking

They tell it to my face that their husband cannot afford such luxury. And you begin to ask yourself what is luxury in eating balanced diet? There is no single plan on malnourishment by the government that I know off. When we notice a child is malnourished, we try to update them on what … to boost the child's health. 9N, GTH.

I have witness on several occasions on my way home, women cooking with firewood having their baby on their back inhaling smokes. 6N, PHC

### Recommendations

#### Policies and strategies

The knowledge of governmental policies differed. Some health workers were not aware of any strategies or access to pneumococcal vaccinations while others had the knowledge and regularly provided vaccination to prevent pneumonia. To eradicate the identified impediment on knowledge and information, some of the participant suggested the need for home delivery services. They further buttressed the matter with already existed services previously carried out on polio that yielded a good result. Some participant suggested the government's intervening before a positive outcome could be attained. They claimed parents can still refuse the nurses vaccinate their children if the government do not make it mandatory.

#### Knowledge of governmental strategies

We don't have any vaccine in our hospital here, neither is there any strategies in place regarding pneumonia that I am aware of. 2N, PH

To my best of knowledge, apart from making the vaccination available in the center, I don't think there is any other plans in place. 3N, PHC

I am hearing it from you for the first time that there is a strategic in place, maybe you educate me about it so I can pass on the information to the mothers when they visit us for help. 14N, PHC

#### Access of vaccination

The vaccines are available in some center. But it's not available here…. the primary health care center, are supposed to carry us along…we receive the highest number of mothers [with sick children] daily at this hospital. 20N, GTH

We don't have any vaccine for pneumonia here, but I know the center where it available. They are supposed to make it available at every center for easy accessibility. 11N, PHC

#### Follow up vaccination

We have the vaccine here, but the turn up is very low. I end up going to some women's house to follow up on them. It's outside my work. But I am human, there are limits to what I can do and to how far I can go. 2N, PHC Will you believe countless numbers of mothers will not return to the clinic as they were instructed? You will only see them again when the situation of the baby is close to giving up. Off course as a mother, I am always emotional. I want to lose my anger on them, but compose myself as professional. 5N, GTH

#### Resolutions

Having discussed barriers faced by the caregivers, regarding vaccinations and attempts to access treatment for children with pneumonia, the participants were requested to share their views on how to solve the problems. They meant that illiteracy and poverty cannot be resolved with health interventions but can be addressed by using local ideas at community level. Some from the rural communities complained about increased poverty due to the killing of farmers and the “Boko Haram's” attacks making it difficult for parents to afford medical bill of their children.

A door-to door service of vaccination was frequently mentioned, which was successful when applied on polio eradication in their community. However, a concern was that parents can still refuse to comply if there is no law for mandatory vaccination. The participants believed the government needs to fund health workers to visit homes and to use media to engage on mass vaccination to educate each household all they need to know about pneumonia.

#### Governmental campaigns

The government imposing law and penalty will take ages, the fastest way is to broadcast the negative impact of not vaccinating your child. This can be done even in radio stations so that everybody will be hearing it. 6PED, GTH The government needs to …provide free education so that the upcoming generation will be educated. The country is rich enough to handle under five pneumonia issues. The government should make available free medical bills on pneumonia related illness. 1PED, GTH

Deaths recorded so far comes after complication. We should rather attack and prevent pneumonia reaching complicated stage. The government should fund us to take the preventive methods to their doorstep. 20N, GTH

#### Household education

The process of door-to-door service was used during polio campaign. Children were vaccinated at their various homes. I think it will make a good impact. 12N, PHC

I just wonder why we should think twice or wait further before implementing home services. I was actively involved in the last polio campaign. The home delivery service was totally effective. All the government need to do is implement the same strategies on pneumonia cases. 21N, PHC

Despite we are operating in a private sector, we can be of help, if the government really want to start educating the parents on pneumonia. I do strongly believe that home service will go a long way. 11N, PH

#### Women will spread the word

If after saving a child of an ignorant mother, she will do the spreading of this news in the community. She will tell her neighbours what she went through for not allowing the vaccine in the first place. They will believe the words of such person compared to you and me. 5N, GTH

## Discussion

Our results show that despite the government's strategies in Nigeria, there are still many unresolved issues that must be addressed before the target of eradicating U5 pneumonia can be achieved. Children were not vaccinated due to lack of vaccines and equipment at health facilities, parent's reluctance to vaccination was recognized and the health personal felt helpless over the situation. Addressing the identified impediments will aid in attaining a progressive and sharp reduction of preventable deaths among the young children in Nigeria. We find that the established strategies are not functioning as planned.

Similar research to ours, was carried out in the North-western part of Nigeria, in March 2020. The results indicated that interventions and strategies alone, may not be sufficient to improve care-seeking and treatment. Perceived and actual poor quality of health services, parents' beliefs about treatment efficacy, social factors, and susceptibility need to be addressed 18. A web-survey in January 2020 explored pediatric stakeholder's views on policy barriers, opportunities, and priorities rules in Nigeria from non-governmental, government, academic, civil society, private, and professional organizations. The result reported lack of pneumonia specific policies, despite acknowledging guidelines that had been adopted 19. Impediments to effective pneumonia management were seen at all levels of the system, from the community to the healthcare policy, with key issues of resourcing centers not fully equipped, and need of improvement of the infrastructure and location of the centers.

In our study, the participants revealed that the financial capacity of the parents was one of the potential factors preventing them from using the health care services. A study conducted in the US among patients of low socioeconomic status found that their participants viewed hospital care as more affordable than primary care. Uninsured patients could not afford the fees for regular primary care visits and relied on charity hospitals when they fell ill. In contrast, our study points out that higher cost of bill was the totality of diagnosing fee, doctor's consultation fees and treatment. Therefore, since the Nigerian healthcare system is completely funded by the patients, this impacts on how recommended health facilities are utilized. It makes it difficult for the parents to access and pay for appropriate healthcare for their children and may consequently lose their child.

Health providers in this study discussed that many children U5 did not receive the recommended vaccination. In addition, children living with remote infrastructure are at more risks of dying of pneumonias compared to those living in urban centres according to mortality studies conducted in Rwanda, Burkina Faso and Bangladesh 20-22. The collected data reveals the complexity and richness of the health providers frustration and helplessness over the unnecessary amount of dead Nigerian children, which could have been saved if they were vaccinated against pneumonia.

## Limitations and strength

There are some limitations in this study. The focus group discussions were conducted in conference rooms, but the rest of the interviews were conducted during working hours on the premises of the health facilities. The short duration of the interviews may have compromised in-depth exploration of the participants' perceptions, especially with the conflicting priority of their normal daily routine. This study was only carried out among health care providers; parents to children were not included. Though it was not the aim of the study, parent's perceptions may differ from the current findings. The present unusual condition of herdsmen-farmer crisis and “Boko haram” killings in the country during the study may have influenced the findings, while the pandemic of COVID-19 was surprisingly not brought up by the participants or discussed.

The concept of reflexivity in this qualitative research acknowledges the input of the several researchers in actively co-constructing the material and sort into categories and use of probing questions added the advantage to elicit specific answers from the participants. Trustworthiness was gained by the collaboration of researchers, one medical doctor with international experiences of qualitative research in low-income countries, one national doctor with experiences of similar research in Nigeria and one performing all data collection, was important to gain a deep understanding of the data. The first author's background and country of origin seems to have influenced the research process in a positive way, in terms of gaining access to health facilities and health personal, the ability of speaking and understanding local dialect and fostering the relationship with the healthcare providers, which all ensured the credibility. The study was performed in a two-month period, which was important for the keeping consistency, and increased the dependability. Transferability was provided by describing the data collection, systematically analysing the data, and illustrating the content by adding important quotes from the participants. This makes it possible for readers to compare to their own context.

## Conclusion

The results from this study highlight several issues regarding the U5 pneumonia mortality in Nigeria, which will need a contextual approach to address them. The findings suggest that factors contributing to these deaths are e.g., lack of knowledge of prevention strategies both among parents, but also among health provider, too late treatment of pneumonia, financial inability and not understanding the importance of vaccination among parents, religion and culture inhibiting seeking health care, far location of health facilities and unequipped health centers.

In order to curtail the above listed impediments, the study proposes resolutions on various levels, such as individual and family education, community involvement and governmental policies to follow and societal campaigns. This means to start a door-to-door service on vaccination program, locate health centers near service users, to enable easy accessibility of health facilities, implementing new policies with mandatory participation in health programs, equipping the health centers with diagnosing tool, available medicine to pediatrician in all centers and increased governmental funding.

## Figures and Tables

**Table 2:** Findings of content analysis of interviews with health professionals, regarding pneumonia among children under five years of age in Nigeria

Theme	Category	Subcategories
Challenges	Anxieties of the current situation	Children are dying unnecessary We are helpless
	Impediments	Poverty and illiteracy Culture and religion Malnourishment and indoor cooking
Recommendations	Policies and strategies	Knowledge of governmental strategies Access to vaccines Follow up vaccinations
	Resolution	Government campaigns Household education Women will spread the word
